# Effectiveness of a multicomponent exercise program in the attenuation of frailty in long-term nursing home residents: study protocol for a randomized clinical controlled trial

**DOI:** 10.1186/s12877-017-0453-0

**Published:** 2017-02-23

**Authors:** Ana Rodriguez-Larrad, Haritz Arrieta, Chloe Rezola, Maider Kortajarena, Jose Javier Yanguas, Miren Iturburu, María Gil Susana, Jon Irazusta

**Affiliations:** 1 Department of Physiology, Faculty of Medicine and Nursing, University of the Basque Country (UPV/EHU), Barrio Sarriena s/n, E-48940 Leioa, Bizkaia Spain; 2 Department of Nursing II, Faculty of Medicine and Nursing, University of the Basque Country (UPV/EHU), J. Begiristain Doktorearen pasealekua 105, E-20014 Donostia-San Sebastian, Gipuzkoa Spain; 3 Matia Instituto Gerontológico, Camino de los Pinos 35, E-20018 Donostia-San Sebastian, Gipuzkoa Spain

**Keywords:** Physical activity, Exercise, Older adults, Aging, Frailty, Cognitive frailty, Nursing-home

## Abstract

**Background:**

There is increasing evidence suggesting that cognition and physical frailty interact within a cycle of decline associated with aging which has been called cognitive frailty. Exercise programs have demonstrated to be an effective tool to prevent functional and cognitive decline during aging, but little is known about their potential to restore or maintain functionality in individuals that require long-term nursing care. Besides, WHO has recently highlighted the importance of introducing systematic musculoskeletal health programs for older people living in residential care, as they represent a particularly vulnerable group for the development of noncommunicable diseases.

**Methods:**

This is a multicentre randomized controlled trial. 114 participants will be randomly allocated to a usual care group or to an intervention group. Inclusion criteria are as follows: ≥ 70 years, ≥ 50 on the Barthel Index, ≥ 20 on MEC-35 who are capable to stand up and walk independently for 10 m. Subjects in the intervention group will add to the activities scheduled for the control group the participation in a 6 months long multicomponent exercise program designed to improve strength, balance and walking retraining. Study assessments will be conducted at baseline and at 3 and 6 months. The primary outcome is change in function assessed by Short Physical Performance Battery and secondary outcomes include other measurements to assess all together the condition of frailty, which includes functionality, sedentary behaviors, cognitive and emotional status and biological markers. The present study has been approved by the Committee on Ethics in Research of the University of the Basque Country (Humans Committee Code M10/2016/105; Biological Samples Committee Code M30/2016/106).

**Discussion:**

Results from this research will show if ageing related functional and cognitive deterioration can be effectively prevented by physical exercise in institutionalized elders. It is expected that the results of this research will guide clinical practice in nursing home settings, so that clinicians and policymakers can provide more evidence-based practice for the management of institutionalized elder people.

**Trial registration:**

The protocol has been registered under the Australian and New Zealand Clinical Trials Registry (ANZCTR) with the identifier: ACTRN12616001044415.

## Background

Globally, older adult population is estimated to reach approximately 22% of the world’s population by 2050 [1, 2] due to the increase in life expectancy. Those older people are characterized by a particularly higher risk of developing negative health-related events because of an age-associated decline in physical and cognitive functions, leading to a progressive disability status. This condition of risk (generally indicated as “frailty”) may support the differentiation of “chronologically” from “biologically” aged individuals in the heterogeneous group of elders [3], and consequently, has emerged as a major clinical and public health priority providing a challenge for health and social care resources development [4].

Otherwise, age-associated frailty is a major concern in geriatrics because of its high prevalence in older persons [5–7] and because it is associated with a greater incidence of disability, hospitalization and death [8]. Although frailty references focus usually on its physical side, there is increasing evidence suggesting that cognition and physical frailty interact within a cycle of decline associated with aging [9]. Actually, affective psychological aspects such as anxiety [10] and depression [11], subjective well-being [12] and quality of life [13, 14] of people are also closely related to frailty.

In this regard, researchers from the International Academy of Nutrition and Aging (IANA) and the International Association of Gerontology and Geriatrics (IAGG) have recently established a definition for “cognitive frailty” in older adults [15]: “an heterogeneous clinical manifestation characterized by the simultaneous presence of physical frailty and cognitive impairment. In particular, the key factors that define such conditions include: 1) the presence of physical frailty and cognitive impairment; and 2) the exclusion of a concurrent clinical diagnosis of Alzheimer disease or other dementias”.

Exercise programs have demonstrated to prevent functional and cognitive decline during aging [16–18]. In the last decade, the study of exercise programs exploring its benefits has been mainly focused on community-dwelling older adults [19], when frailty is identified at an early stage. When compared with control interventions, physical exercise programs have shown to reverse frailty and improve cognition, emotional, and social networking in controlled populations of community-dwelling frail older adults [20, 21]. Otherwise, while it is widely accepted that frailty can be considered reversible at early stages, mild to moderate disability has proven to be hardly reversible by interventions at old age [22], when individuals require long-term nursing care.

In spite of the widely known health benefits associated with physical activity, older adults represent a very sedentary behavior cohort [23, 24]. Sedentary behavior refers to any waking activity characterized by low energy expenditure (1.0 to 1.5 basal metabolic rate) and a sitting or reclining posture [25]. There is a new body of evidence centered on the negative impact of sedentary behaviors for health, which links it with a higher risk of cardiovascular disease, metabolic syndrome, obesity, and other negative health outcomes, independent of physical activity levels, among older adults [26, 27]. Furthermore, several studies have demonstrated the association between the sedentary behaviors and the development of functional limitations in older adults [28–35]. Nevertheless, little is known about the associations of sedentary behavior with variables that are important for successful aging including mental [36, 37], cognitive [38], biological markers [39] and quality of life indicators [40–42].

Finally, about 60 different potential biomarkers of frailty have been postulated, most of them involved in inflammation, oxidative stress and metabolism which affect different organ systems [43, 44]. Inflammation appears to play a major role in the pathophysiology of frailty; in fact, a positive relationship between frailty-related indexes and markers of inflammation has been observed [45]. Several studies have also detected higher serum levels of interleukin 6 (IL-6), CRP and IL-1Ra in fragile patients, which have been associated with lower muscle strength and a slower gait [46–48]. On the other hand, brain-derived neural factor (BDNF) which is related to brain plasticity and function, has demonstrated to be influenced by physical exercise [49]. Despite this evidence, nowadays there is no clear consensus about the validity of such biomarkers in primary and hospital care and they are not commonly used for identifying frailty in clinical settings.

## Objective

To the knowledge of the authors, no studies have explored the effects of a supervised multicomponent exercise program carried out in long-term nursing care centers from a broad perspective of the condition of frailty, assessing all together functionality, sedentary behaviors, cognitive and emotional status and biological markers. Thus, it has been designed a randomized multicenter study to test the hypothesis that the addition of a multicomponent exercise program to the usual care in institutionalized elders can improve their functionality in 1 point in the Short Physical Performance Battery (SPPB).

The major aim of this study is to ascertain if a supervised multicomponent exercise program carried out in long-term nursing care centers improves or maintains functionality, sedentary behaviors, cognitive and emotional status, health related quality of life and modifies biological markers related to frailty when compared with a control population that received usual care.

The present study is based on a previous pilot study [50] in which we successfully collected preliminary data to accurately demonstrate the feasibility of recruitment, estimate the required sample size for the current trial, confirm the adherence and safety of the intervention, refine the outcome assessments, and optimize the organizational infrastructure.

## Methods

### Study design and participants

**Fig. 1:**
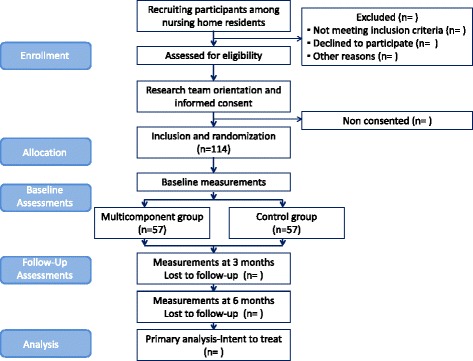
Study protocol description

With the above mentioned objective in mind, it has been designed an experimental multicentre simple randomized study, with random allocation to a usual care group or to an intervention group. Each site will enroll on average 15 subjects. Researchers responsible for data gathering will be blinded for this study. Participants will be recruited from Matia and Caser Residential Care Facilities in 7 long-term nursing homes (San Sebastian, Basque Country, Spain). It is expected that the intervention will take place between October 2016 and June 2017. Study assessments will be conducted by blinded research staff during clinic visits at baseline, as well as at 3 and 6 months from the beginning of the intervention. The CONSORT Statement extension for trials of non-pharmacological interventions and pragmatic intervention trials has been used to design the study and will be used to report it (Fig. 1).

### Inclusion and exclusion criteria

Subjects will be considered eligible for the study if they fulfill all of the following criteria: aged ≥ 70 years, scored ≥ 50 on the Barthel Index [51], scored ≥ 20 on MEC-35 [52] Test (an adapted and validated version of Mini Mental State Examination (MMSE) in Spanish) who are all capable to stand up and walk independently for at least 10 m.

Participants will not be eligible for the study if they are clinically unstable under the clinical judgment of the medical professionals of the reference center, or in any other condition that means that entering the study would not be in the subject’s best interests.

### Recruitment and randomization

The listing of individuals that meet inclusion criteria will be obtained from the database of Matia and Caser Residential Care Facilities. The primary recruitment strategy will be information provided to the potential participants by the medical and nursing professionals from each nursing home. All the volunteers will receive detailed study information in their reference sites through the research team: objectives, measurement variables and other details about the interventions will be explained orally and in writing, to both potential participants and their families. Informed consent will be obtained from each participant who will sign it after fully understanding the procedures. Afterwards they will be randomly assigned (1:1 ratio) by center through sealed opaque envelopes to either the control or the intervention group by coin-tossing sequence generation.

### Control group

Subjects in the control group will participate in the routine activities that all nursing homes usually offer to the attendees: memory workshops, reading, singing, etc. Activities will be low intensity in any case.

### Multicomponent exercise program

Subjects in the intervention group will add to the activities scheduled for the control group, the participation in a multicomponent exercise program designed to improve strength, balance and walking retraining conducted by an experienced physical trainer. Strength and balance training will be performed through supervised sessions, while walking retraining will be carried out through individualized recommendations that participants will fulfill on their own. The technical content of the program is based on a specific literature review [17, 53, 54] including authors’ expertise and field experience, and it is divided into two sections of 3 months long (Table 1). Each section has specific objectives and a standardized framework (combination and sequence of exercises), but the goals are individualized based on each participants’ level of physical fitness. Goals will be adapted in response to illness, injury or physical symptoms. The intervention has been designed to meet the exercise and physical activity guidelines for older adults established by the American College of Sport Medicine (ACSM) and American Heart Association (AHA) [55, 56]. Training attendance will be recorded every session.

**Table 1:** Multicomponent exercise program’s technical content

	3 MONTHS			3 MONTHS		
	Familiarisation phase			Development phase		
	Objective: Increase strength			Objective: Improve functional capacity		
	1^ST^ MONTH	2^ND^ MONTH	3^RD^ MONTH	4^TH^ MONTH	5^TH^ MONTH	6^TH^ MONTH
Strength	3–4 Ex: 1–2 sets, 8–12 rep at 40% of 1RM	4 Ex: 2 sets, 8–12 rep at 50% of 1RM	4–5 Ex: 2 sets, 8–12 rep at 60% of 1RM	4–5 Ex: 3 sets, 8–12 rep at 60–65% of 1RM	4–5 Ex: 2 sets, 7–8 rep at 70% of 1RM	3 Ex: 1 set,7–8 rep at 70% of 1RM
Balance	2–3 exercises, progressive difficulty in sitting position and decreasing arm support when standing position.			4–5 exercises, progressive difficulty in standing position, decreasing arm support, increasing instability and external perturbations.		
Walking program	5 min WR 5 days (M)	10 min WR 5 days (5′ M and 5′ A)	14 min WR 5 days (7′ M and 7′ A)	18 min WR 5 days (9′ M and 9′ A)	22 min WR 5 days (11′ M and 11′ A)	22 min WR 7 days (11′ M and 11′ A)

*Ex* exercises, *rep* repetitions, *WR* walking recommendation, *M* morning, *A* afternoon

Forty-five min supervised sessions directed to improve strength and balance will be conducted twice a week. An interval of at least 48 h between training sessions will be respected (Table 2). All sessions will begin with a brief warm-up of 5 min (range-of-motion exercises for the neck, wrists, shoulders, hip, knees and ankles). Strength training (25 min) will comprise upper and lower body exercises performed with external weights, which will be tailored to the individual’s functional capacity through Brzycki equation for the estimation of 1-RM (repetition maximum) at baseline and at the end of every month, to ensure an appropriate training stimulus. In all strength tests subjects will be encouraged verbally to perform each exercise as forcefully as possible in a standardized form. In the three first months exercises will be performed with light loads (40–60% 1-RM) to ensure an appropriate adaption to resistance exercise and thereafter loads, if they are well tolerated, will be increased to 65–70% 1-RM for additional benefits.

**Table 2:** Programation of the intervention for the 13th week

Objective	Monday	Tuesday	Wednesday	Thursday	Friday	Saturday	Sunday
Multicomponent exercise program							
Warm-Up 5 min		Range of motion for different joints		Range of motion for different joints			
Strength training 25 min	-	Arm curl 60% 3 sets 8-12rep	-	Arm curl 60% 3 sets 8-12rep	-	-	-
	-	Chair stand 60% 3 sets 8-12rep	-	Chair stand 60% 3 sets 8-12rep	-	-	-
	-	Leg flexion 60% 3 sets 8-12rep	-	Leg flexion 60% 3 sets 8-12rep	-	-	-
	-	Leg abduction 60% 3 sets 8-12rep	-	-	-	-	-
	-	-	-	Hip extensión 60% 3 sets 8-12rep	-	-	-
	-	Standing on tips and heels 3 sets 10 rep	-	Standing on tips and heels 3 sets 10 rep	-	-	-
Balance training 10 min	-	One legged stand 2 sets 10 s	-	One legged stand 2 sets 10 s	-	-	-
	-	Semi-tandem/Tandem exercises 2 sets 10 s	-	Semi-tandem/Tandem exercises 2 sets 10 s	-	-	-
	-	Circuit training 2 sets	-	Circuit training 2 sets	-	-	-
	-	Stepping 2 sets 10 rep	-	Stepping 2 sets 10rep	-	-	-
	-	Ball reaching 2 sets	-	-	-	-	-
Cooling-Down 5 min		Stretching, breathing, relaxing exercises.		Stretching, breathing, relaxing exercises.			
Walking retraining							
	9 min WR M & A	-	9 min WR M & A	-	9 min WR M & A	9 min WR M & A	9 min WR M & A

*WR* walking recommendation, *M* morning, *A* afternoon

Balance training (10 min) will include exercises in progressing difficulty starting by decreasing arm support (with 2 arms at first, with one hand, and finally none if possible) along with decreasing base of support (both feet together, semi-tandem and tandem positions) and increasing complexity of movements as to challenge participants’ balance as they progress. Exercises will be varied through the period: weight transfer from one leg to another, walking with small obstacles, proprioceptive exercises and stepping practice. Sessions will finish with 5 min of cooling down by stretching, breathing and relaxing exercises.

Walking retraining will also be implemented through individualized recommendations regarding distance and intensity to perform on their own in addition to the supervised sessions. According to ACSM/AHA guidelines [56], exercise intensity will be monitored using a category-ratio 0–10 scale for physical exertion and breathlessness (Borg CR10 scale) [57]. Participants will be instructed to walk at a moderate intensity, equivalent to a 5–6 on the CR10 scale, with a target goal of achieving at least 22 min/day at the end of the 6 months period. Walking retraining will initially begin with light intensity activity for short periods of time, which gradually will be increased in intensity and duration over the 6 months period.

Finally, attendance to the program may be suspended due to a hospitalization, injury, or any other health events. Evaluation for re-engaging the exercitation will depend on the functional impact of the illness and on any activity limitation prescriptions that may provide the participant’s health care team. Irrespective of the week of the intervention that a suspension may occur, all restarts will be conducted in a supervised and progressive way.

### Outcome measures

The primary outcome measure will be the difference in function between intervention and control group assessed by changes in summary ordinal score on the Short Physical Performance Battery [58] (SPPB). SPPB consists of three tests: balance, gait ability and leg strength. The score for each test is given in categorical modality (0–4) based on run time intervals, and the total score will range from 0 (worst) to 12 points (best). The SPPB has been shown to be a valid instrument for screening frailty and predicting disability, institutionalization and mortality. A total score of less than 10 points indicates frailty and a high risk of disability and falls. 1 point change in the total score has demonstrated to be of clinical relevance [59, 60].

The following parameters will be also recorded: age, gender, socioeconomic situation, marital status, Barthel index [51], MEC-35 [52], Lubben Social Network Scale (LSNS-6) [61], Tilburg Frail index [62], Frailty index [63], and Charlson [64] index. Anthropometric data will include body mass index (BMI) and waist-hip ratio; fat mass percentage will be measured using a portable bio-electrical impedance analyzer (Bodystat BIA Quadscan 4000) [65].

Functional examination will include the following (Table 3): Senior Fitness Test [66], instrumented Timed Up and Go test [67] (iTUG; BTS Biomedical G-WALK triaxial accelerometer and gyroscope), comfortable and fast walking speed [68], bilateral handgrip strength test [69] (Jamar dynamometer), Berg balance test [70], static balance and fall-risk by stabilometer [71] (Biodex Balance System SD), as well as active and sedentary periods during everyday life recorded with an accelerometer (Actigraph GT3X model (Actigraph LLC, Pensacola, FL, USA)) that will be worn on the hip with a belt for a 7 day period. The device will be set to quantify the number of steps taken per day. In line with that, active-period intensities will be classified following the criteria developed by Freedson et al. [72] as light, moderate or vigorous intensity and measured in minutes performed in each intensity.

**Table 3:** Functional assessment tests

Test (Reference)	Functions/Parameters	Description
Short Physical Performance Battery (SPPB) [58]	Lower extremity function: static balance, gait speed and getting in and out of a chair	Side-by-side, semi-tandem and tandem stands (10 s); 4 meters walk test at comfortable speed and 5 quickly sit to stand from a chair without upper extremity assistance
Senior Fitness Test [66]	Upper and lower extremity strength and flexibility, static and dynamic balance and aerobic capacity	Chair-stands in 30 s; 6-min walking test; arm curl test (30 s); chair sit and reach; back scratch and 8 foot up and go test
Instrumented Timed Up and Go test (BTS Biomedical G-WALK) [67]	Dynamic balance	Get up from a chair, walk 3 meters at a normal pace, turn and walk back to sit down again
Instrumented walking speed (BTS Biomedical G-WALK) [68]	Standard gait parameters: speed, step frequency, cadence	Walk for 4 and 10 meters at comfortable and fast speed
Bilateral handgrip strength test (Jamar dynamometer) [69]	Hand grip strength	Squeez the dynamometer with maximum isometric effort for about 5 s
Berg balance test [70]	Postural stability	Performance of 14 functional tasks
Stabilometry (Biodex Balance System) [71]	Ability to control balance on an oscillatory platform	Two-legged stance counterbalancing of an standardized oscillatory platform displacements
Accelerometry (Actigraph GT3X model (Actigraph LLC, Pensacola, FL, USA) [72]	Active and sedentary periods during everyday life	7 days period quantification of the number of steps performed per day and minutes completed at light, moderate or vigorous intensity

Cognitive and emotional assessment will be determined by the following (Table 4): Clinical Dementia Rating [73] (CDR), Montreal Cognitive Assessment (MoCA) [74], Symbol Digit Modalities Test (SDMT) [75] and Anxiety and Depression Goldberg Scale [76]. Health related quality of life will be assessed by the questionnaire EQ-5D-5 L [77].

**Table 4:** Cognitive and Functional assessment tests

Test (Reference)	Functions	Description
Clinical Dementia Rating (CDR) [73]	Cognitive and functional performance	Covered domains: Memory, Orientation, Judgment and Problem Solving, Community Affairs, Home and Hobbies, Personal Care
Montreal Cognitive Assessment (MoCA) [74]	Mild Cognitive Impairment, Early Alzheimer’s disease	Covered domains: Attention and Concentration, Executive Functions, Memory, Language, Visuoconstructional Skills, Conceptual Thinking, Calculations, Orientation
Symbol Digit Modalities Test (SDMT) [75]	Cognitive impairment	Covered domains: Attention, Visual Scanning, Motor Speed
Anxiety and Depression Goldberg Scale [76]	Affective state	Includes nine depression and nine anxiety items from the past month
Questionnaire EQ-5D-5 L [77]	Health related quality of life	Self-rated quality of life related to health; included dimensions: Mobility, Self-Care, Usual Activities, Pain/Discomfort, Anxiety/Depression

Blood samples will be obtained and stored at − 80 °C. Biomarkers will be measured according to standard laboratory protocols at the Physiology laboratory in the University of the Basque Country using an ELISA kit (ChemiKine TM; Millipore, Temecula, CA) following the manufacturer’s instructions. Thus, myostatin [78], irisin [79], interleukin 6 [46] and BDNF [49] will be measured (Table 5).

**Table 5:** Biomarkers that will be analysed in the study

Biomarker (Reference)	Associated Function
Myostatin [78]	Miokine associated to the muscle gain inhibition
Irisin [79]	Miokine associated to the increase of thermogenesis with physical activity
Interleukin 6 [46]	Inflammatory marker associated to frailty and physical performance
Brain-Derived Neural Factor (BDNF) [49]	Neurotrophic factor associated to cognitive function

Finally, we will also record the number of falls, visits to the emergency service, hospital admissions and length of hospital stay.

### Safety assessments

All co-existing diseases or conditions related with the intervention will be treated in accordance with prevailing medical practice and will be reported as an adverse event.

### Power and sample size

Sample size has been calculated to detect minimal significant effects on the variable of physical performance (SPPB) [80, 81]: accepting an alpha risk of 0.05 and a beta risk of 0.20 in a bilateral contrast, 86 individuals are required in order to detect a difference equal to or greater than 1 unit in the SPPB (SD = 2.34). It has been increased the sample size in an additional 20% (loses during follow-up) and 5% (mortality). The resultant sample size is determinate in 114 individuals, therefore 57 individuals per group (intervention and control group).

### Statistical considerations

The IBM SPSS Statistics 23 statistical software package (SPSS, Inc., Chicago, IL) will be used to analyse the data. Intention to treat analyses will be performed. The normal distribution of the data will be evaluated using the Kolmogorov-Smirnov test. Continuous variables will be expressed as mean (SD) when normally distributed and as median with interquartile range (IQR) when not. Categorical variables will be expressed as frequency counts and percentages. Statistical comparisons at baseline will be performed using appropriate statistical tests according to the type and distribution of the data: *t* test or Mann–Whitney *U*-test for continuous variables and Chi-squared test for categorical variables. The intervention-related effects will be performed using appropriate statistical tests according to the type and distribution of the data: an analysis of variance (ANOVA) or Friedman test with repeated measures (0, 3 and 6 months). When a significant F value is obtained, LSD post hoc procedures will be used to evaluate pairwise differences. *p* < 0.05 will be considered to be statistically significant. Furthermore, an analysis of covariance (ANCOVA) will be done to compare the data between intervention and control groups, considering as co-variables baseline measurements, as well as other variables as age or gender.

### Trial status

The trial is currently being set up with participant recruitment. Recruitment will cease when 114 participants have been randomized; it is expected this target will be reached by June 2017.

## Discussion

This is a multicenter study designed to ascertain if a supervised multicomponent exercise program carried out in long-term nursing care centers improves or maintains functionality, sedentary behaviors, cognitive and emotional status and biological markers related to frailty when compared with a control population that receives usual care. To our knowledge, an exercise program carried out in nursing home elderly population has not been studied before from a so broad perspective, taking into account all together functional, cognitive, emotional and biochemical conditions. The current lack of definitive evidence on whether ageing related functional deterioration can be effectively prevented by physical exercise in institutionalized elders represents a potential obstacle to the development of guidelines for geriatric clinicians and policymakers that would also report in increasing health-related quality of life for a prevalent and clinically-relevant population. Furthermore, the World Health Organization (WHO) has recently published an Action Plan for the Prevention and Control of Noncommunicable Diseases in the WHO European Region 2016–2025, where it is highlighted the importance of introducing systematic musculoskeletal health programs for older people, including those living in residential care [82]. Moreover, long-term nursing home residents have been identified as a particularly vulnerable group where the above mentioned plan should direct its actions through an early intervention to restore and maintain functionality.

The exercise program that is described in this protocol has been designed to be feasible, easy to implement and potentially delivered in any nursing home settings, which may have direct clinical applications. We previous reported [83] that a similar multicomponent exercise program is feasible, well tolerated and pleasantly welcomed by individuals living in long-term care facilities. Furthermore, improvements in functional status were observed in those participants that took part in the program, particularly in gait ability, balance and aerobic capacity. These findings are in line with other studies carried out in nursing homes, indicating that the exercise programs can benefit functional performance, well being and cognition of the residents [84–88]. Nevertheless, to date few randomized clinical trials have been conducted in institutionalized elders, and normally these trials study heterogeneous interventions (sometimes poorly explained), while our study allows the extrapolation of results and the implementation of the program to any other nursing home through a well-defined methodology.

Finally, if the described multicomponent exercise program proves to report benefits in terms of functional, sedentary behavior, cognitive and emotional status, as well as knowledge on the response of biological markers to physical activity, the findings could provide evidence suggesting the need to augment the standard physical practice prescribed at nursing homes in the elder population. Otherwise, failure to reject the null hypothesis would suggest that the progression of the decline associated with the aging process in at-risk persons continues on to disability, despite any potential benefits from physical activity. This would be an important study outcome as well and implies that efforts to hold back the process of disablement in this population should be directed elsewhere.

The study of whether multicomponent exercise program can improve or maintain functionality, sedentary behaviors, cognitive and emotional status and biological markers related with frailty in nursing home elders is nowadays an unanswered question that is of major importance to public health and social policy. It is expected that the results of this research will guide clinical practice in nursing home settings, so that clinicians and policymakers can provide more evidence-based practice for the management of institutionalized elder people.

## Abbreviations

ACSM: American College of sport medicine
AHA: American heart association
ANZCTR: Australian and New Zealand clinical trials registry
BDNF: Brain-derived neural factor
BMI: Body mass index
CDR: Clinical dementia rating
CONSORT: Consolidated Standards of reporting trials
CRP: C-Reactive protein
EQ-5D-5 L: EuroQol five-dimensional questionnaire
IAGG: International association of gerontology and geriatrics
IANA: International academy of nutrition and aging
IL-1Ra: Interleukin 1 reception antagonist
IL-6: Interleukin 6
IQR: InterQuartile range
iTUG: Instrumented timed up and go test
LSNS: Lubben social network scale
MEC-35: Cognition mini-exam of lobo
MoCA: Montreal cognitive assessment
RM: Repetition maximum
SDMT: Symbol digit modalities test
SPPB: Short physical performance battery
WHO: World health organisation
